# MAPS; acute safety data of the St Jude accent - tendril IPG system during prolonged max power CMR scanning

**DOI:** 10.1186/1532-429X-17-S1-M6

**Published:** 2015-02-03

**Authors:** Mark P Ainslie, Anna Reid, Christopher A Miller, David Clark, Benjamin Brown, David Fox, Neil Davidson, Andrew Trafford, Matthias Schmitt

**Affiliations:** 1 Cardiology, UHSM, Manchester, UK; 2 University of Manchester, Manchester, UK

## Background

Until recently, the use of MRI in patients with PPM's was prohibited. The lifetime probability of a patient with a cardiac device requiring an MRI is 50-75%. Serious adverse events occurring during MRI of patients with cardiac devices are rare, but include asystole, VF and death. There is a clinical need to develop knowledge of MR safe devices and safe scanning protocols.

This study specifically address these issues in the SJM Accent MR PPM System, undergoing a dedicated CMR scan at 1.5 T, with a long scan duration at max power.

## Methods

Patients were recruited into the MAPS trial and implanted with a SJM Accent ppm and 2 tendril MR leads. All patients were PPM dependent. CMR was performed more than 6 weeks following implant. Pacing capture thresholds, impedances and battery voltages were assessed prior to, between and immediately following the CMR scan. The scans were performed on a Siemens Avanto 1.5T scanner. All patients were placed in an MR pacing mode. Each scan duration was over 90 mins.

## Results

Between February 2012 and February 2014, 50 patients were implanted with the SJM MR ppm. The mean age of the patients was 67.3±8.1 years, 30 male. All 50 patients had at least 1 CMR.

There were no significant adverse events reported during any of the scans and no loss of capture was seen in any scan.

### Pacing thresholds

The mean pacing threshold for RVOT lead at implant was 0.67±0.22V and at 2 week check was 0.73±0.21V. Pacing thresholds prior to the 1^st^ CMR scan, between the lead switch over and following the scan were 0.66±0.16V, 0.66±0.16V and 0.69±0.27V respectively, p=0.34.

The mean pacing threshold for the apical lead at implant was 0.71±0.29V and at 2 week check was 0.74±0.26V. Pacing thresholds prior to the 1^st^ CMR scan, between the lead switch over and following the scan were 0.69±0.17V, 0.69±0.16V and 0.69±0.16V respectively, p= 1.

### Impedance

The mean pacing impedance for the RVOT lead at implant was 739±168Ω and at 2 week check was 655±251Ω. Pacing impedances prior to the CMR scan, between the lead switch over and following the scan were 601±123Ω, 595±114Ω and 579±141Ω respectively, p=0.008.

The mean pacing impedance for Apical lead at implant was 631±130Ω and at 2 week check was 616±81Ω. Pacing impedances prior to the CMR scan, between the lead switch over and following the scan were 612±81Ω, 611±80Ω and 574±69Ω respectively, p=0.004.

### Battery

The mean battery voltage prior to, between and following every CMR scan did not alter acutely. CMR scan 1 was at 2.99±0.03V.

Specific absorption rate

**Figure 1:**
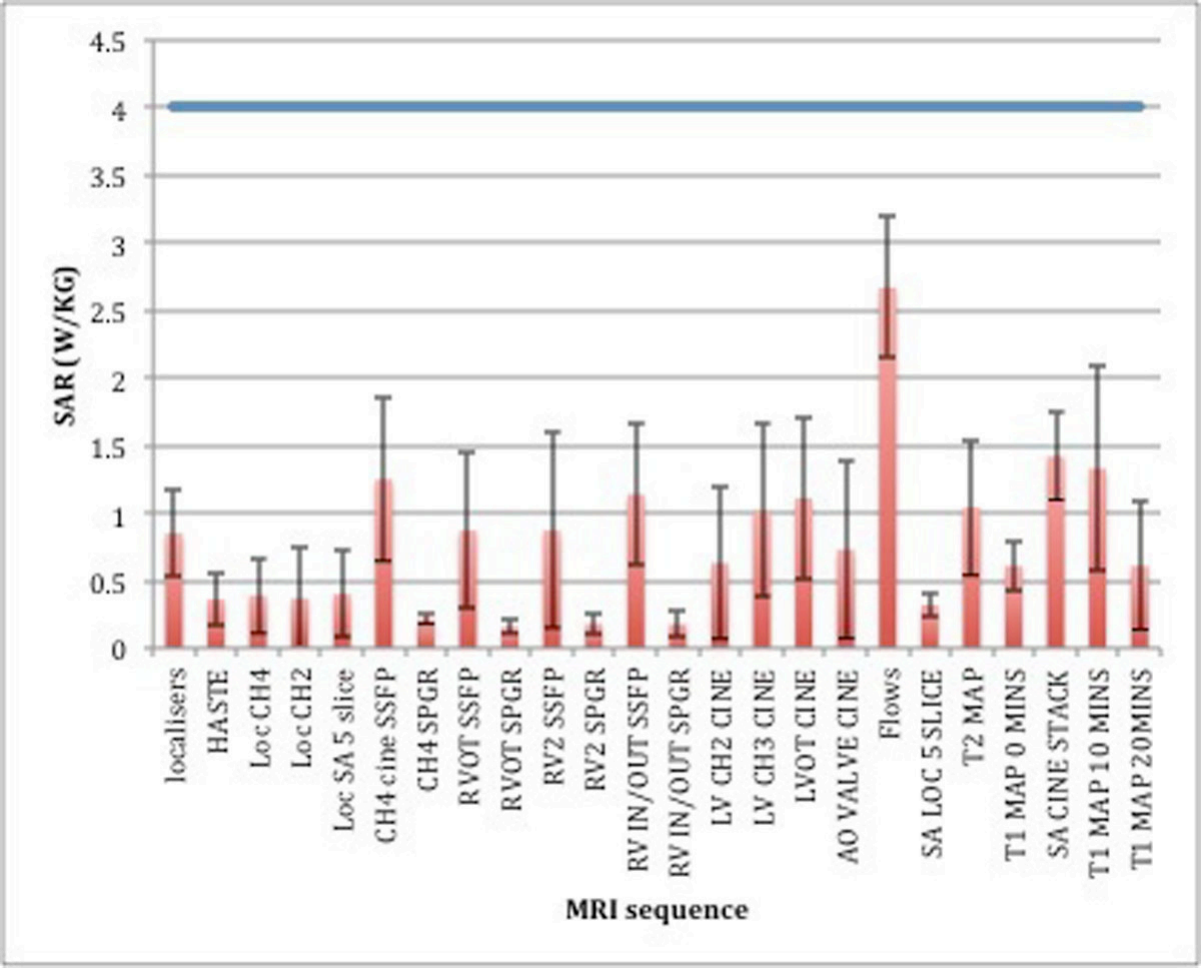
illustrates the Mean SAR of the different MR sequences for 10 scans.

The max SAR of 4 w/kg was never exceeded. See Fig 1.

Tendril leads 5 control patients had the MR system but no CMR scans. Table 1 compares the parameters between cohorts over 12 months. A similar trend in parameter changes was observed between the CMR and non-CMR pacing cohort.

**Table 1:** Control group of 5 patients with MR system but no CMR scans compared to study group.

		0 months	2 months	12 months
Threshold (V)	Control	0.625 +/-0.14	0.6±04	0.65±0.3

	CMR group	0.67±0.16	0.67±0.2	0.7±0.48

Impedance Ω	Control	698±135	568±85	537±92

	CMR group	739±167	585±108	558±154

Battery (V)	Control	2.99	2.99	2.96

	CMR group	3	3	2.96

## Conclusions

Prolonged max power CMR scanning of the St Jude Accent - Tendril IPG system at 1.5 T is safe and has no clinically relevant effects on PCT, voltage and Battery power.

## Funding

British Heart Foundation St Jude Fellowship Grant.

